# Caffeine Consumption and Rhinologic Symptom Severity

**DOI:** 10.1002/ohn.70329

**Published:** 2026-06-30

**Authors:** Duncan G. J. Green, Steven X. Wang, Michael S. DeBakey, Nrusheel Kattar, Edward D. McCoul

**Affiliations:** ^1^ Department of Otorhinolaryngology Ochsner Health New Orleans Los Angeles USA; ^2^ Department of Otolaryngology–Head and Neck Surgery Tulane University New Orleans Los Angeles USA; ^3^ Department of Otolaryngology–Head and Neck Surgery Louisiana State University Shreveport Los Angeles USA; ^4^ Ochsner Clinical School University of Queensland New Orleans Louisiana USA

**Keywords:** addiction, caffeine, rhinitis, rhinology, sinusitis

## Abstract

Caffeine is the most widely consumed habit‐forming drug in the world. A relationship has been proposed between caffeine consumption and rhinologic symptoms. To determine whether the potential deleterious effects of caffeine withdrawal are outweighed by the rhinologic symptom benefits, our group conducted a prospective study of patients presenting with rhinologic complaints at local clinics. Rhinologic symptom severity was assessed using the SNOT‐22 and the frequency and quantity of daily caffeine consumption was assessed by questionnaire. A total of 226 participants completed the study. No correlation was found between caffeine consumption and total SNOT‐22 (*P* > .05), rhinologic SNOT‐22 subscale (*P* > .05), or tertiles of low, medium, and high caffeine consumers (*P* = .18). This suggests that caffeine consumption does not have an appreciable effect on rhinologic symptoms.

Caffeine is the most consumed habit‐forming drug in the world yet has been subject to limited study in otolaryngology. A potential relationship has been proposed between consumption of caffeinated beverages and rhinologic symptoms, with greater consumption associated with greater symptom severity.^1^ The putative mechanism behind this effect is unclear. Caffeine has been suggested to influence olfaction, increasing odor sensitivity in nonusers.^2^ Cessation of caffeine is known to have unpleasant withdrawal symptoms, including headaches, irritability, difficulty concentrating, and nausea.^3^

A growing interest in the effect of diet and lifestyle modifications on aspects of health suggests a potential role for caffeine abstinence as a management strategy for rhinologic symptoms. There is also concern that caffeine consumption may be an overlooked confounding variable in clinical study design. Therefore, we sought to evaluate the association between the quantity of daily caffeine consumption and severity of rhinologic symptoms.

## Methods

A cross‐sectional study was performed on consecutive new adult patients presenting to the outpatient otolaryngology practice of the senior author (EDM) between June 2020 and June 2021. At the start of the visit, each participant was asked to complete the Sino‐Nasal Outcome Test (SNOT‐22) assessment. SNOT‐22 subdomain scores were calculated (nasal, extranasal, ear/face, psychological, sleep).^4^ Caffeine use habits were obtained from an itemized survey of daily consumption of coffee, tea, caffeinated soda, and energy drink. For each of the 4 beverages, the number of drinks per day and the number of days per week were recorded. From this data, a weekly consumption estimate was calculated, represented by mg/day of caffeine. Weekly consumption was converted to milligrams of caffeine per week using the most common caffeine concentrations per United States Department of Agriculture (USDA) guidelines.^5^ Caffeine per drink was determined via USDA guidelines to be 71 mg per 6‐oz coffee, 35.6 mg per 6‐oz black tea, 47.2 mg per caffeinated soda, and 144 mg per energy drink. Participants who did not complete both forms were excluded. Participants were stratified into 3 consumption groups based on approximate tertiles: none or minimal use (0‐50 mg/day), moderate use (50‐175 mg/day), and high use (>175 mg/day). Power analysis indicated that, with an assumed correlation of *r* ≥ 0.185 and *α* = 0.05 (two‐tailed), 80% would be achieved by approximately 200 participants. Given the absence of prior research examining the effect of caffeine on rhinologic symptoms, this small effect size threshold was considered appropriate for exploratory analysis.

Univariate analyses of nonnormally distributed SNOT‐22 scores and subgroup scores were conducted using Spearman's rank regression and dose‐response regression in R (R Foundation for Statistical Computing). Analyses of between‐group differences were conducted using one‐way analysis of variance and *t*‐tests to measure top and bottom deciles. Directed acyclic graph (DAG) analysis was conducted using the DAGitty toolbox to explore multivariate relationships between caffeine use and multiple subscores. Data have been visualized using GraphPad Prism. This study was approved by the institutional review board of Ochsner Medical Center.

## Results

**Figure 1:**
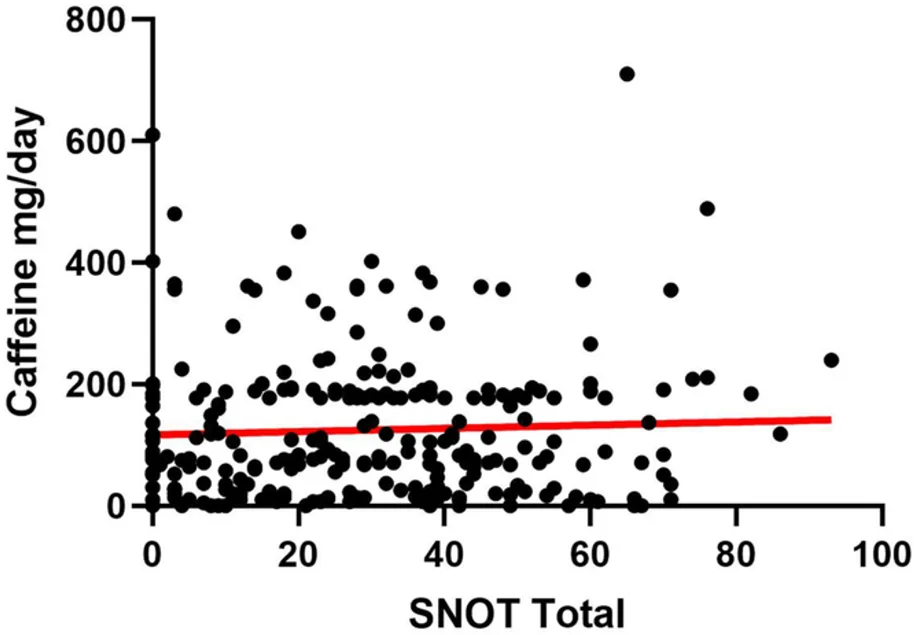
Spearman's rank regression of mg/day of caffeine and total rhinological symptom score (*P* = .23). SNOT‐22, Sino‐nasal outcome test.

**Figure 2:**
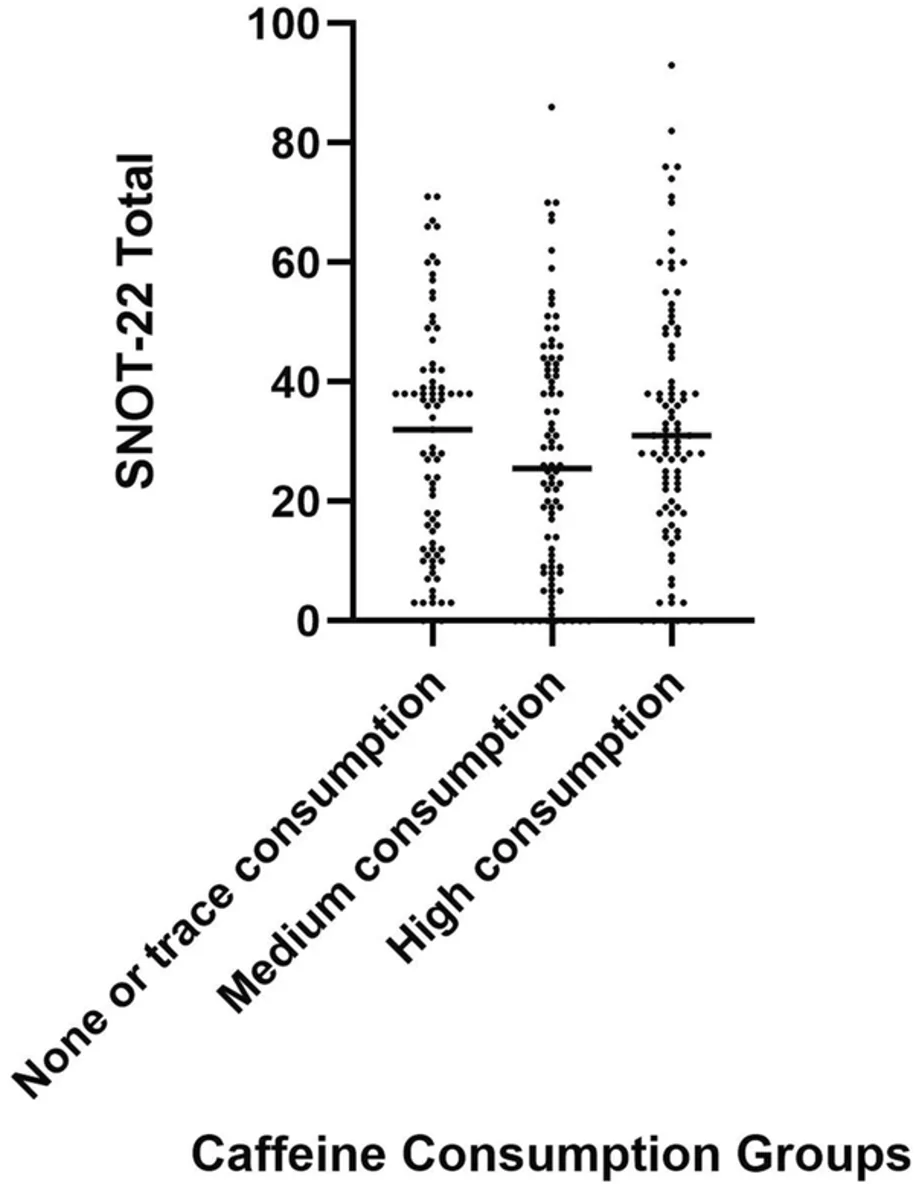
One‐way ANOVA of caffeine consumption groups and total rhinological symptom score (*P* = .18). ANOVA, analysis of variance; SNOT‐22, sino‐nasal outcome test.

226 participants were included for analysis. The sample was 57.1% (129) female with a mean age of 36.4 years. Mean (SD) weekly caffeine consumption was 127.8 (116.7) mg. The first tertile median (SD) weekly caffeine consumption was 13.5 (12.1) mg, second tertile was 82.8 (29.1) mg, and third was 192.9 (102.2) mg. No significant association was found between estimated daily caffeine consumption and SNOT‐22 total or subscores (nasal, extranasal, ear/face, psychological, sleep) (all *P* > .05) (Figure 1). Further analysis of consumption tertiles revealed no effect of caffeine use on SNOT‐22 scores (*P* = .18) (Figure 2). No relationship was found between caffeine use and sex or age for SNOT‐22 total or subscores (*P* > .05), which was unchanged by controlling for age or sex in primary outcome analyses.

DAG analysis found no interaction between caffeine consumption, total SNOT‐22 or SNOT‐22 subscores in any path. Further exploratory analysis to probe this nonsignificance found no clinically meaningful difference of SNOT‐22 total or subscores between the top 10% of caffeine consumers and the bottom 10% of caffeine consumers (mean difference = 4.3, *d* = 0.35, *P* > .05). A dose‐response analysis found minimal effect of caffeine consumption on total SNOT‐22 scores (0.8 increase in SNOT‐22 scores per 100 mg caffeine/day).

Further analyses of patient characteristics found no difference in caffeine use between patients who presented with allergic symptoms and those that did not (*t* = 0.65, *P* > .05). Furthermore, there was no association between caffeine use in patients that are reported decongestant use and those that did not (*t* = 0.52, *P* > .05).

## Discussion

No meaningful relationship was found between consumption of caffeinated beverages and rhinologic symptoms as reflected by SNOT‐22 total and subdomain scores. These findings suggest that caffeine use may not contribute to the presentation of rhinologic symptoms and likely do not need to be considered as a confounding variable in future studies. Clinically, these findings do not support a recommendation for patients to abstain from caffeine to mitigate rhinologic symptoms.

It is possible that we were unable to find an effect in our sample due to the overall low use of caffeine. Approximately one‐third of participants consumed no or minimal caffeine on a weekly basis. Further, only 27 participants in our sample reported caffeine consumption above 250 mg/day, while 10 participants exceeded the USDA maximum recommended intake (400 mg/day).^5^ An effect specific to high caffeine users may exist which this study was underpowered to detect. Future studies examining the effect of caffeine on rhinologic symptoms specific to high caffeine users may be informative. Furthermore, larger, more comprehensive studies could examine the interaction between environmental irritants, caffeine use, and rhinologic symptoms.

In conclusion, caffeine consumption does not appear to have an appreciable effect on rhinologic symptoms. The potential deleterious effects of caffeine withdrawal, especially among high caffeine consumers, would not be outweighed by the expected benefit.

## Author Contributions

**Duncan G. J. Green**, design, analysis, drafting, final approval, accountable; **Steven X. Wang**, analysis, drafting, final approval, accountable; **Michael S. DeBakey**, design, data collection, critical revision final approval, accountable; **Nrusheel Kattar**, design, data collection, critical revision, final approval, accountable; **Edward D. McCoul**, conception, design, analysis of research, drafting, final approval, accountable.

## Disclosures

### Competing interests

EDM is a consultant for 3D Matrix, Advanced Rx, Sanofi/Regeneron, and Zsquare. The other authors have nothing to disclose.

### Funding source

This work is supported by a grant from the EENT Foundation (EE231102).
